# Research and instruction services for online advanced practice nursing programs: a survey of North American academic librarians

**DOI:** 10.5195/jmla.2019.689

**Published:** 2019-10-01

**Authors:** Gregg A. Stevens, Elizabeth G. Hinton, Roy E. Brown

**Affiliations:** Senior Assistant Librarian/Health Sciences Librarian, Health Sciences Library, Stony Brook University, Stony Brook, NY, gregg.stevens@stonybrook.edu; Reference Librarian, Rowland Medical Library, University of Mississippi Medical Center, Jackson, MS, ehinton@umc.edu; Research and Education Librarian, Tompkins-McCaw Library, Virginia Commonwealth University, Richmond, VA, rebrown2@vcu.edu

## Abstract

**Introduction:**

The increasing popularity of distance education has led many advanced practice nursing (APN) programs to shift to either online or hybrid models. To meet the needs of these students, some nursing librarians are using technology for virtual research and instruction. This study was designed to assess the extent to which librarians in North America are providing virtual research and instruction services for APN students.

**Methods:**

An institutional review board–approved, ten-question survey was developed to determine how librarians are providing services for APN students. It was announced in October 2017 through several health sciences librarian email discussion lists. The survey ran for four weeks. Data were analyzed using Qualtrics and Excel.

**Results:**

Eighty complete responses were received. The majority of respondents (66%) indicated that their universities’ APN programs were conducted in a hybrid format. Sixty-seven percent also indicated that they provide library instruction in person. Most librarians indicated that they have provided research assistance through some virtual method (phone or email, at 90% and 97%, respectively), and some have used online chat (42%) and video chat (35%). A strong majority of librarians (96%) indicated that they felt comfortable using technology to provide research assistance and instruction.

**Conclusion:**

Opportunities exist to leverage technology to provide virtual research assistance and instruction. Greater promotion of these alternative methods can supplement traditional in-person services to provide greater flexibility for graduate nursing students’ busy schedules. Some outreach may be necessary to highlight the advantages of virtual services, and further research is needed to identify other barriers and potential solutions.

## INTRODUCTION

Advanced practice nurses (APNs)—which include nurse practitioners (NPs), nurse anesthetists, nurse midwives, and clinical nurse specialists (CNSs)—are increasingly important health care providers in North America who help fill shortages in primary care, especially in rural and other underserved areas [1]. Allowing nurses with advanced training to take on some of the responsibilities of physicians not only reduces the impact of physician shortages, but also improves the quality and efficiency of the health care system by providing comparable services at a lower cost [2]. In Canada, the number of NPs has tripled since 2006, with most specializing in primary care or family practice. The role of NPs has also become more standardized across provinces, so that the basic functions of independent diagnosis and prescription are in the scope of practice of all Canadian NPs [3]. In the United States, the scope of practice of NPs is limited by different state legislatures. As of 2015, twenty-two states, four territories, and the District of Columbia give NPs full practice authority, allowing them to diagnose and treat patients independently of a physician [4]. Regardless of their scope of practice, however, APNs play vital roles in health care delivery across North America [1, 2].

To create an APN workforce, many North American nursing colleges and universities offer graduate degrees in advanced practice. In 2013, 420 institutions offered some type of APN program in the United States, representing 51.6% of the institutional members of the American Association of Colleges of Nursing (AACN) [5, 6]. In 2016, 28 Canadian schools offered at least 1 APN program, representing 20.4% of all nursing schools in the country [7].

The average graduate nursing student is demographically very different from the average undergraduate nursing student and, therefore, has different educational needs and preferences. As graduate nursing students are generally registered nurses who might work nights and weekends [8], an asynchronous education format can allow students to complete programs with some schedule flexibility. Graduate nursing students are often returning to school after several years of working in the field and, thus, are older than undergraduate nursing students [8]. According to the National League of Nursing, 13% of undergraduate bachelor’s degree in nursing (BSN) students in 2015–2016 were over 30 years old, whereas 45% of master’s degree students and 82% of doctoral nursing students were over 30 years old [9]. Additionally, as more APN programs are offered online, geographic proximity to campus is becoming less of a factor for potential APN students. Research indicates that rural students are more likely to enroll in online APN programs than in on-site programs [10].

Therefore, in-person graduate nursing education and accompanying library instruction may be becoming less commonplace. Also, distance learning—which puts less emphasis on lectures and more emphasis on active learning, individual and group projects, and critical thinking—also fits with the shift in pedagogical style in nursing education from a teacher-centered model to a student-centered model [11]. Librarians have traditionally been educational partners for nursing students and faculty, teaching concepts such as critical thinking and project research, which help to complement the nursing curriculum.

As APN programs migrate into distance format, nursing librarians who serve these programs should shift their reference and instruction services online. In its *Standards for Distance Learning Library Services*, the Association for College and Research Libraries (ACRL) states that “library services offered to the distance learning community must be designed to meet a wide range of informational, instructional, and user needs, and should provide some form of direct user access to library personnel,” including online services for instruction, research, and consultation [12]. The accrediting bodies of nursing programs such as the National League for Nursing Accrediting Commission (NLNAC) and the AACN also mention the need to provide library services to all nursing students regardless of their location [13].

To provide equitable services for distance students, librarians can employ technologies to offer reference and instruction services online. Reference services can be offered synchronously via telephone, chat, or video-conferencing software as well as asynchronously through email, and instruction services can be provided synchronously through video conferencing as well as asynchronously through recorded materials. Both reference and instruction can also be conducted through a learning management system (LMS), such as Blackboard.

A review of the existing literature provides limited information on how librarians have provided services for online graduate nursing programs. In a report on liaison librarian work with graduate nursing students in distance programs at the University of Kansas Medical Center, Whitehair described providing limited in-person instruction during orientations but in-depth research instruction through video conferencing software. Also, reference services were provided to individuals through a variety of modes: in person, online conferencing, instant messaging, and phone [14]. Gebb and Young of Frontier Nursing University, which offers online APN programs, described an in-person instruction session focused on mobile resources for point-of-care during a multiday clinical intensive [15]. Several other articles described how nursing librarians have embedded resources and services into their nursing schools’ LMSs. Guillot et al. and Sullo et al. reported embedding instruction and reference services for graduate-level nursing courses in Blackboard [16, 17], and other authors reported similar efforts with undergraduate nursing students [18–20].

However, the authors found no published studies that sought to determine the prevalence of online reference and instruction services offered by librarians working with graduate nursing programs across North America. To address this gap, we aimed to determine the extent to which nursing liaison librarians in the United States and Canada have moved their reference and instruction services for APN students online as their programs have transitioned into hybrid or fully online formats.

## METHODS

To determine the extent to which librarians provide reference and instruction services for APN distance programs, we created a ten-question survey (supplemental Appendix A). No personal information was obtained by the survey beyond respondents’ geographical locations. The Stony Brook University Institutional Review Board assessed the project and exempted it from further review (CORIHS 2017-4216-F).

The survey was administered through Stony Brook University’s Qualtrics account [21] in October 2017 and was open for 4 weeks. We publicized the survey through several mailing lists that health sciences librarians commonly use: the Medical Library Association’s (MLA’s) MEDLIB-L, the Canadian Health Libraries Association/Association des bibliothèques de la santé du Canada’s CANMEDLIB, the email discussion lists of 4 MLA chapters (Southern, Mid-Atlantic, New York-New Jersey, and Philadelphia Regional), and MLA’s Nursing and Allied Health Resources Section email discussion list. As MEDLIB-L alone has approximately 1,700 subscribers and CANMEDLIB-L has more than 400 subscribers, we believed that we were able to reach most North American health sciences librarians through this distribution method. Additionally, we promoted the survey through social media by making several announcements on one author’s personal Twitter account using the #medlibs hashtag, which many health sciences librarians follow.

After the survey closed, the complete data set was exported from Qualtrics to a CSV file. This spreadsheet was used in Microsoft Excel to allow easier sorting of the responses and the creation of separate datasets for the United States and Canada [22].

## RESULTS

Of the 105 survey respondents, 90 indicated that their institution had an APN program, allowing them to proceed with the remainder of the survey. Ten of these 90 respondents did not fully complete the survey. Therefore, a total of 80 respondents completed the survey. Most (n=71) respondents were from the United States, and fewer (n=9) were from Canada. The respondents represented 35 states and provinces, with California and Massachusetts being the most well-represented states (supplemental Appendix B). Based on the previously cited total number of schools with APN programs (420 in the United States and 28 in Canada), this represented a de facto response rate of 17% of schools in the United States and 32% of schools in Canada.

Nearly all respondents (98%) indicated that their institutions offered an NP program (supplemental Appendix C). Additionally, 30% of respondents were from schools with a nurse anesthetist program, 30% from schools with a CNS program, and 19% from schools with a nurse midwife program. Some (15%) respondents also indicated that their schools offered another type of APN program. Regarding degrees granted by the APN programs, 81% and 83% of respondents indicated that their schools offered doctoral and master’s degrees, respectively; 36% indicated that their schools offered a graduate APN certificate; and 5% indicated that their schools offered another type of degree.

Most (66%) respondents indicated that their institution’s APN program was offered in a hybrid format, 16% were from schools with traditional in-person classes for the APN programs, 9% stated that their schools’ programs were completely online, and 9% stated that they were not sure of the format offered at their schools. For this question, we found a substantial difference between Canadian and US respondents. More than half of the Canadian respondents (56%) indicated that the APN programs at their schools were offered in traditional in-person format, 22% stated that their schools’ programs were in hybrid format, and 11% indicated that their school’s program was entirely online. The US responses for this question were 11% traditional, 71.8% hybrid, and 8.5% completely online.

Most respondents indicated that they offered traditional reference services to APN students during the previous year, with 97% offering email reference services, 91% offering in-person reference services (drop-in or by appointment), and 90% offering phone reference services. Less than half of respondents stated that they provided some form of online chat (42%) or video chat (35%) reference services. When asked about the most common form of reference service used by students, most respondents indicated in-person (40%) or email (42%) reference, whereas fewer respondents indicated video chat (6%), phone (5%), or online chat (4%) reference.

When asked about instruction provided to APN students, most respondents (81%) indicated teaching in-person classes, whereas fewer taught an online class with video-conferencing software (35%) or taught through some form of chat without audio or video (e.g., chat room, 14%). Some respondents (10%) had not taught any APN classes within the past year. When asked about the most common format for instruction, two-thirds indicated that they generally taught in person. The librarians indicating online chat (1%) or online video (10%) as their most common instructional format represented a much smaller fraction of those responding.

When asked if their discomfort with technology was a barrier to offering online reference or instruction services, nearly all respondents (96%) expressed comfort with the requisite technology, with only 4% of respondents indicating they were slightly uncomfortable and none reporting moderate or extreme discomfort. Most respondents (83%) stated that they felt extremely or moderately comfortable with the requisite technology.

## DISCUSSION

Our results reveal what seems to be a contradiction in how nursing librarians provide instruction services to graduate nursing students. With 75% of respondents indicating that their institutions offered APN programs either completely online or in a hybrid format, one might expect that the modalities of reference and instruction services that librarians offer would be compatible with distance education. Although this seemed to be true for reference services, with most respondents regularly providing email and phone reference and a fair number providing video or chat reference, two-thirds of respondents indicated that they most commonly provided instruction in-person, not online. This demonstrates a disconnect between best practices for distance library services and the frequent reality of in-person instruction. One could speculate that this is the result of librarian discomfort with online instruction technologies; however, the vast majority of respondents stated that they did not perceive any personal barriers to using online technology for reference and instruction.

Our survey results suggest that online reference is fairly common for graduate nursing programs. One hypothesis as to why it is not more pervasive is that APN students may opt for in-person research assistance. Librarianship could still be considered by many users to be a face-to-face business, and student insecurity with using databases and crafting detailed search strategies could lead to more requests for in-person appointments. Depending on the technology available to the librarian and the student, adequate demonstrations of the proper use of databases might be impossible without meeting in person. Further, both the librarian and the student could find it more beneficial to teach and learn in person.

Because distance students use an LMS such as Blackboard or Canvas for their coursework, it is unlikely that many APN students would experience discomfort with working online. Thus, the low use of online reference services may reflect a student preference for in-person consultations. Because we only surveyed nursing librarians, further research would be needed to determine if APN students perceive any barriers to online library services or prefer in-person services over those offered remotely. Previous research in nursing education indicates that the number of students citing technology as a barrier to their learning is relatively small but significant [8].

Another possible explanation for the continued high use of in-person library services is that many librarians who serve hybrid APN programs provide most of their services when the students are on campus. Some librarians may only provide in-person instruction as a component of new student orientation on campus and not later in the curriculum when students are actually working on research projects and no longer on campus. Yet another possibility is that the instruction is being scheduled on regular “on-site” days, vying for time in the day’s schedule and for student attention with other activities, such as clinical skills practice and group discussions. In both of these possible scenarios, in-person librarian sessions may simply be a continuation of traditional modes of teaching.

This legacy of in-person instruction could be considered a potential institutional barrier to online instruction. Whitehair addressed this challenge by stressing the importance of using orientation sessions to promote research services so that students can seek out reference assistance later. She also reported teaching research skills to DNP students at the point of need (i.e., during their capstone course) through online synchronous classes [14]. Likewise, Gebb and Young’s in-person instruction session on mobile apps takes advantage of one of the few times that their APN students physically visit the campus [15].

Librarians who utilize online tools to provide instruction and develop educational materials could take advantage of opportunities to provide instruction when it is more appropriate in the curriculum as well as make that instruction more meaningful to students. Also, a transition to an online presence at a later point in the curriculum would require strong relationships with the APN nursing faculty. Future research could attempt to identify the systemic barriers to librarian involvement with their liaison units and how this could affect the transition from in-person to online instruction for APN programs.

Regarding the number of “don’t know” responses to the question asking about the APN programs at the respondent’s institution, we note several possible explanations. One possibility is that these respondents were new librarians who had not yet fully integrated into their institutions, and therefore, they were not completely familiar with their schools’ programs. Another possibility is that the librarians did not work closely enough with their schools’ curricula to know which programs were offered. Some librarians might have been from understaffed library environments, serving as liaisons to several academic units, so their involvement with APN programs could have been limited. Finally, librarians who were not nursing liaisons could have attempted to take the survey and, thus, would not have been familiar enough with the APN programs at their institutions to provide adequate answers.

As the survey was offered to nursing librarians in both the United States and Canada, we separated out the nine Canadian responses to assess any differences in library services for APN program services between the two countries. The only major difference involved the Canadian responses to the question about the format of the APN programs. If these nine responses were representative of Canadian nursing education, it seems that Canadian APN programs have not shifted online at the same rate as similar programs in the United States, rather they are offered more often in person at Canadian schools. However, the responses on how Canadian librarians provided reference and instruction services to their APN programs were similar to those of their US counterparts. This might indicate that despite differences in the education and health care systems between the two countries, librarians in the United States and Canada work similarly with APN students and faculty.

Due to the good response rate of US nursing librarians (n=71) and decent geographic saturation of respondents, our results might serve as sufficient basis to suggest that improvements could be made in the extent of online reference and instruction services for APN programs. Due to the relatively low response rate of Canadian librarians, however, further research with a larger number of participants would be needed to better gauge the state of distance services for APN students in Canada.

There are a number of limitations to this study. One is that our self-selected group of respondents is not necessarily representative of nursing librarians across North America. Our survey announcement in the email discussion lists of four Eastern MLA chapters and the name recognition of the authors might have contributed to a higher response rate from states in the Eastern and Southeastern United States, resulting in a possible overrepresentation of those states. Consistent with this possibility, there was a lower than anticipated response from Midwestern and Western states and provinces relative to the overall population and number of nursing schools in these areas.

Also, because we did not ask for institutional affiliations, it is possible that more than one librarian from the same school answered the survey, creating overrepresentation of certain schools. Several “other” and “not sure” responses may also be perceived as problematic. We acknowledge that providing a free-text box could have prompted more descriptive responses to several survey items, such as the types of APN programs and degrees offered and additional methods of reference and instruction. Without more information, we can only speculate as to what “other” modes of reference and instruction were used by respondents. Further, due to the anonymous nature of the survey, follow-up with respondents was impossible. Future research on this subject could allow respondents to provide email addresses if they are willing to answer further questions about individual responses.

The mixed results, diversity of program formats, and individual institutional circumstances indicate that we cannot recommend any single method of library reference and instruction for all nursing librarians. However, our survey was designed to be exploratory and, thus, represents an important first step in gauging the degree to which distance library services are being implemented in North American APN programs. With further research on how librarians are providing these services and which modalities are most successful, it is possible that a set of competencies could be identified for those who provide services to nursing programs in the online environment. Furthermore, a toolbox of best practices could be developed to meet these competencies, which could include evidence-based recommendations for providing quality online instruction and reference services as well as faculty outreach suggestions. Such tools would support MLA’s professional competency for Instruction and Instructional Design, which encourages the use of technologies to improve instruction and communication [23].

Based on the results of this exploratory study, we are considering exploring how librarians can further improve their online research and instruction services. Two avenues under consideration are surveying APN students regarding their opinions about library services and another assessing the effectiveness of strategies used to integrate online instruction into the APN curriculum. Any next steps will be done with multi-institutional partners to increase the sample size and strengthen the data set, which will allow conclusions that are drawn to be more generalizable.

Due to the evolving educational environment for APN programs, librarians must continuously promote themselves. Adapting to the virtual environment is one opportunity for working with students who might otherwise be underserved. Effective outreach to both students and faculty is crucial. Through social media, online research guides, or promotion during in-person interactions, librarians should be proactive in marketing the services that they offer. Successful marketing should translate to increased engagement while demonstrating the value of librarians to their respective schools of nursing and institutions as a whole.

## SUPPLEMENTAL FILES

Appendix A Questionnaire

Appendix B Geographic distribution of respondents completing full survey (survey question 1)

Appendix C Survey results: questions 2–10

## 

**Gregg A. Stevens, AHIP**, gregg.stevens@stonybrook.edu, http://orcid.org/0000-0003-4088-6742, Senior Assistant Librarian/Health Sciences Librarian, Health Sciences Library, Stony Brook University, Stony Brook, NY

**Elizabeth G. Hinton, AHIP**, ehinton@umc.edu, http://orcid.org/0000-0002-3793-2683, Reference Librarian, Rowland Medical Library, University of Mississippi Medical Center, Jackson, MS

**Roy E. Brown, AHIP**, rebrown2@vcu.edu, http://orcid.org/0000-0002-0627-9132, Research and Education Librarian, Tompkins-McCaw Library, Virginia Commonwealth University, Richmond, VA
