# Supplementary material for: Research and instruction services for online advanced practice nursing programs: a survey of North American academic librarians
Source: J Med Libr Assoc. 2019 Oct 1;107(4):508–14. doi: 10.5195/jmla.2019.689 (PMC6774546; doi:10.5195/jmla.2019.689)
Supplement: Appendix A [file jmla-107-508-s001.pdf]

Journal of the Medical Library Association

Supplemental content to
J Med Libr Assoc. Oct;107(4):dx.doi.org/10.5195/jmla.2019.689
www.jmla.mlanet.org
© Stevens, Hinton, Brown 2019

Research and instruction services for online advanced practice nursing programs: a survey of North American academic librarians

Gregg A. Stevens, AHIP; Elizabeth G. Hinton, AHIP; Roy E. Brown, AHIP

APPENDIX A

Questionnaire

Research Consent Form

Project Title: Advanced Practice Nursing Survey

Principal Investigator: Gregg A. Stevens, AHIP

You are being asked to be a volunteer in a research study.

Purpose: The purpose of this study is to assess the level of librarian involvement with advance practice nursing (APN) programs in the United States and Canada.

Procedures: If you decide to be in this study, your part will involve a short ten-question survey on your university's APN programs and your work with those programs.

Risks/Discomforts: There are no foreseeable risks or discomforts associated with your participation in this study.

Benefits: There is no direct benefit expected as a result of you being in this study.

Credit to Subjects: You will not be paid for your participation.

Confidentiality: All the information we get about you will be not be linked to you at all. We will do this by not writing down your name or anything else that could link you in any way to the answers you give us for our study. All the study data that we get from you will be kept locked up. If any papers and talks are given about this research, your name will not be used.

Costs to You: You will not incur any costs to participate in this study.

Alternatives: Your alternative to being in this study is to simply not participate.

Your Rights as a Research Subject: Your participation in this study is voluntary. You do not have to be in this study if you do not want to be. You have the right to change your mind and leave the study at any time without giving any reason and without penalty. Any new information that may make you change your mind about being in this study will be given to you. You can print a copy of this consent form. You do not lose any of your legal rights by participating in this study.

Questions about the Study or Your Rights as a Research Subject:

- If you have any questions, concerns, or complaints about the study, you may contact Gregg Stevens at 631.444.3102.
- If you have any questions about your rights as a research subject or if you would like to obtain information or offer input, you may contact the Stony Brook University Research Subject Advocate, Ms. Lu-Ann Kozlowski, BSN, RN, 631.632.9036, OR by email, lu-ann.kozlowski@stonybrook.edu.
- Visit Stony Brook University's Volunteering in Research page for more information about participating in research, frequently asked questions, and an opportunity to provide feedback and comments or ask questions related to your experience as a research subject.

JMLA Journal of the Medical Library Association

SC
Supplemental content to
J Med Libr Assoc. Oct;107(4):dx.doi.org/10.5195/jmla.2019.689
www.jmla.mlanet.org
© Stevens, Hinton, Brown 2019

If you complete the following survey, it means that you have read (or have had read to you) the information given in this consent form, and you would like to be a volunteer in this study.

Q1. Where is your university located? (Choose your state, territory, or province)

- o Alabama
- o Alaska
- o Arizona
- o Arkansas
- o California
- o Colorado
- o Connecticut
- o Delaware
- o District of Columbia
- o Florida
- o Georgia
- o Hawaii
- o Idaho
- o Illinois
- o Indiana
- o Iowa
- o Kansas
- o Kentucky
- o Louisiana
- o Maine
- o Maryland
- o Massachusetts
- o Michigan
- o Minnesota
- o Mississippi
- o Missouri
- o Montana
- o Nebraska
- o Nevada
- o New Hampshire
- o New Jersey
- o New Mexico
- o New York
- o North Carolina
- o North Dakota
- o Ohio
- o Oklahoma
- o Oregon
- o Pennsylvania
- o Rhode Island
- o South Carolina
- o South Dakota
- o Tennessee
- o Texas
- o Utah
- o Vermont
- o Virginia
- o Washington
- o West Virginia
- o Wisconsin
- o Wyoming
- o American Samoa
- o Guam
- o Northern Mariana Islands
- o Puerto Rico
- o US Virgin Islands
- o Alberta
- o British Columbia
- o Manitoba
- o New Brunswick
- o Newfoundland and Labrador
- o Northwest Territories
- o Nova Scotia
- o Nunavut
- o Ontario
- o Prince Edward Island
- o Quebec
- o Saskatchewan
- o Yukon
- o My university is not in the US or Canada

Q2. Does your university offer advanced practice nursing (APN) program(s)?

- o Yes
- o No
- o Not sure

JMLA Journal of the Medical Library Association

Supplemental content to
J Med Libr Assoc. Oct;107(4):dx.doi.org/10.5195/jmla.2019.689
www.jmla.mlanet.org
© Stevens, Hinton, Brown 2019

Q3. What types of APN programs does your university offer? (Select all that apply)

- Nurse practitioner (any type)
- Nurse midwife
- Nurse anesthetist
- Clinical nurse specialist (CNS)
- Other

Q4. What degree(s) does your university offer for APN? (Select all that apply)

- Doctorate
- Master's
- Graduate certificate
- Other

Q5. Does your university offer APN programs in a distance format?

- Yes, all classes are completely online
- Yes, mostly online, but occasionally students have to come to campus (hybrid format)
- No, students have all classes physically on campus
- Not sure

Q6. What types of research service (answering basic questions, providing consultations) have you provided to APN students within the past year? (Select all that apply)

- In person (drop-in or by appointment)
- Email
- Phone
- Online chat (no audio or video)
- Video chat
- Other
- I haven't provided any research services this past year for APN students

Q7. Of the contact methods mentioned above, which one has been the most common? (Select one)

- In person (drop-in or by appointment)
- Email
- Phone
- Online chat (no audio or video)
- Video chat
- Other
- I haven't provided any research services this past year for APN students

JMLA Journal of the Medical Library Association

Supplemental content to
J Med Libr Assoc. Oct;107(4):dx.doi.org/10.5195/jmla.2019.689
www.jmla.mlanet.org
© Stevens, Hinton, Brown 2019

Q8. What types of instruction services have you provided for APN classes? (Select all that apply)

- In person
- Online chat with no audio or video (e.g., chat room in Blackboard)
- Online class with video conferencing software (e.g., WebEx)
- Other
- I haven't taught any APN classes this past year

Q9. Of the instruction methods mentioned above, which one has been the most common? (Select one)

- In person
- Online chat with no audio or video (e.g., chat room in Blackboard)
- Online class with video conferencing software (e.g., WebEx)
- Other
- I haven't taught any APN classes this past year

Q10. How comfortable do you feel using online technology to provide instruction and research services?

- Extremely comfortable
- Moderately comfortable
- Slightly comfortable
- Neither comfortable nor uncomfortable
- Slightly uncomfortable
- Moderately uncomfortable
- Extremely uncomfortable
