# Supplementary material for: Research and instruction services for online advanced practice nursing programs: a survey of North American academic librarians
Source: J Med Libr Assoc. 2019 Oct 1;107(4):508–14. doi: 10.5195/jmla.2019.689 (PMC6774546; doi:10.5195/jmla.2019.689)
Supplement: Appendix B [file jmla-107-508-s002.pdf]

JMLA Journal of the Medical Library Association

Supplemental content to
J Med Libr Assoc. Oct;107(4):dx.doi.org/10.5195/jmla.2019.689
www.jmla.mlanet.org
© Stevens, Hinton, Brown 2019

Research and instruction services for online advanced practice nursing programs: a survey of North American academic librarians

Gregg A. Stevens, AHIP; Elizabeth G. Hinton, AHIP; Roy E. Brown, AHIP

APPENDIX B

Geographic distribution of respondents completing full survey (survey question 1)

Q1. Where is your university located? (Choose your state, territory, or province)

State	Number of responses	Province	Number of responses
California	6	Ontario	3
Massachusetts	5	Quebec	2
Georgia	4	Alberta	1
Kentucky	4	Manitoba	1
Michigan	4	Newfoundland and Labrador	1
Pennsylvania	4	Nova Scotia	1
Tennessee	4		
Alabama	3		
Illinois	3		
New York	3		
North Carolina	3		
Arkansas	2		
Florida	2		
Indiana	2		
Mississippi	2		
Missouri	2		
Ohio	2		
Texas	2		
Virginia	2		
Washington	2		
Wisconsin	2		
Colorado	1		
Connecticut	1		
Louisiana	1		
Maryland	1		

JMLA Journal of the Medical Library Association

SC
Supplemental content to
J Med Libr Assoc. Oct;107(4):dx.doi.org/10.5195/jmla.2019.689
www.jmla.mlanet.org
© Stevens, Hinton, Brown 2019

State	Number of responses	Province	Number of responses
Nevada	1		
New Jersey	1		
South Carolina	1		
Utah	1		
Total US responses	71	Total Canadian responses	9
Total all responses	80		
