# Supplementary material for: Research and instruction services for online advanced practice nursing programs: a survey of North American academic librarians
Source: J Med Libr Assoc. 2019 Oct 1;107(4):508–14. doi: 10.5195/jmla.2019.689 (PMC6774546; doi:10.5195/jmla.2019.689)
Supplement: Appendix C [file jmla-107-508-s003.pdf]

JMLA Journal of the Medical Library Association

Supplemental content to
J Med Libr Assoc. Oct;107(4):dx.doi.org/10.5195/jmla.2019.689
www.jmla.mlanet.org
© Stevens, Hinton, Brown 2019

Research and instruction services for online advanced practice nursing programs: a survey of North American academic librarians

Gregg A. Stevens, AHIP; Elizabeth G. Hinton, AHIP; Roy E. Brown, AHIP

APPENDIX C

Survey results: questions 2–10

Q2. Does your university offer advanced practice nursing (APN) program(s)?

#	Answer	%	Count
1	Yes	85.71%	90
2	No	10.48%	11
3	Not sure	3.81%	4
	Total	100.00%	105

Q3. What types of APN programs does your university offer? (Select all that apply)

#	Answer	%	Count
1	Nurse practitioner (any type)	97.50%	78
2	Nurse midwife	18.75%	15
3	Nurse anesthetist	30.00%	24
4	Clinical nurse specialist (CNS)	30.00%	24
5	Other	15.00%	12
	Total		153
	Respondents		80

Q4. What degree(s) does your university offer for APN? (Select all that apply)

#	Answer	%	Count
1	Doctorate	81.25%	65
2	Master's	82.50%	66
3	Graduate certificate	36.25%	29
4	Other	5.00%	4
	Total		164
	Respondents		80

JMLA Journal of the Medical Library Association

SC
Supplemental content to
J Med Libr Assoc. Oct;107(4):dx.doi.org/10.5195/jmla.2019.689
www.jmla.mlanet.org
© Stevens, Hinton, Brown 2019

Q5. Does your university offer APN programs in a distance format?

#	Answer	%	Count
1	Yes, all classes are completely online	8.75%	7
2	Yes, mostly online, but occasionally students have to come to campus (hybrid format)	66.25%	53
3	No, students have all classes physically on campus	16.25%	13
4	Not sure	8.75%	7
	Total	100.00%	80

Q6. What types of research service (answering basic questions, providing consultations) have you provided to APN students within the past year? (Select all that apply)

#	Answer	%	Count
1	In person (drop-in or by appointment)	91.03%	71
2	Email	97.44%	76
3	Phone	89.74 %	70
4	Online chat (no audio or video)	42.31%	33
5	Video chat	34.62%	27
6	Other	21.79%	17
7	I haven't provided any research services this past year for APN students	1.28%	1
	Total	100.00%	78

Q7. Of the contact methods mentioned above, which one has been the most common? (Select one)

#	Answer	%	Count
1	In person (drop-in or by appointment)	39.74%	31
2	Email	42.31%	33
3	Phone	5.13%	4
4	Online chat (no audio or video)	3.85%	3
5	Video chat	6.41%	5
6	Other	1.28%	1
7	I haven't provided any research services this past year for APN students	1.28%	1
	Total	100.00%	78

JMLA Journal of the Medical Library Association

Supplemental content to
J Med Libr Assoc. Oct;107(4):dx.doi.org/10.5195/jmla.2019.689
www.jmla.mlanet.org
© Stevens, Hinton, Brown 2019

Q8. What types of instruction services have you provided for APN classes? (Select all that apply)

#	Answer	%	Count
1	In person	80.77%	63
2	Online chat with no audio or video (e.g., chat room in Blackboard)	14.10%	11
3	Online class with video conferencing software (e.g., WebEx)	34.62%	27
4	Other	24.36%	19
5	I haven't taught any APN classes this past year	10.26%	8
	Total	100.00%	78

Q9. Of the instruction methods mentioned above, which one has been the most common? (Select one)

#	Answer	%	Count
1	In person	66.67%	52
2	Online chat with no audio or video (e.g., chat room in Blackboard)	1.28%	1
3	Online class with video conferencing software (e.g., WebEx)	10.26%	8
4	Other	11.54%	9
5	I haven't taught any APN classes this past year	10.26%	8
	Total	100.00%	78

Q10. How comfortable do you feel using online technology to provide instruction and research services?

#	Answer	%	Count
1	Extremely comfortable	39.74%	31
2	Moderately comfortable	43.59%	34
3	Slightly comfortable	11.54%	9
4	Neither comfortable nor uncomfortable	1.28%	1
5	Slightly uncomfortable	3.85%	3
6	Moderately uncomfortable	–	0
7	Extremely uncomfortable	–	0
	Total	100.00%	78
